# The Neuroprotective Effects of *Coreopsis tinctoria* and Its Mechanism: Interpretation of Network Pharmacological and Experimental Data

**DOI:** 10.3389/fphar.2021.791288

**Published:** 2022-02-11

**Authors:** Pei Ma, Rong Zhang, Lijia Xu, Haibo Liu, Peigen Xiao

**Affiliations:** ^1^ Institute of Medicinal Plant Development, Chinese Academy of Medical Sciences, Peking Union Medical College, Beijing, China; ^2^ Key Laboratory of Bioactive Substances and Resources Utilization of Chinese Herbal Medicine, Ministry of Education, Beijing, China

**Keywords:** *Coreopsis tinctoria*, neurodegenerative disease, apoptosis, AKT signaling, network pharmacology

## Abstract

**Background:** *Coreopsis tinctoria* Nutt. (CT), an annual herb in the genus *Coreopsis*, is an important traditional medicine to be used for antidiabetes and antioxidation.

**Objective:** The antioxidant compounds from CT may affect mitochondrial function and apoptosis, which in turn may affect related diseases. The aim of this study was to explore the potential molecular mechanism and new therapeutic opportunities of CT based on network pharmacology.

**Methods:** A network pharmacology-based method, which combined data collection, drug-likeness filtering, target prediction, disease prediction, and network analysis, was used to decipher the potential targets and new therapeutic opportunities of CT. The potential molecular mechanism and pathway were explored through Gene Ontology (GO) and KEGG analyses. Then MPTP-induced SH-SY5Y cell model was applied to evaluate the neuroprotective effects and key targets.

**Results:** There were 1,011 targets predicted for 110 compounds. Most targets were regulated by flavones, phenylpropanoids, and phenols and had synergistic effects on memory impairment, pancreatic neoplasm, fatty liver disease, and so on. The compounds–targets–diseases network identified TNF, PTGS2, VEGFA, BCL2, HIF1A, MMP9, PIK3CG, ALDH2, AKT1, and EGFR as key targets. The GO and KEGG analyses revealed that the cell death pathway, mitochondrial energy metabolism, and PI3K-AKT signal pathway were the main pathways. CT showed neuroprotective effects via regulating gene and protein expression levels of key targets in an *in vitro* model.

**Conclusion:** CT had potential neuroprotective effects by targeting multiple targets related with apoptosis, which were affected by the BCL-2 and AKT signaling pathways. This study provided a theoretical basis for the research of neuroprotective effects of CT.

## Introduction

Neurodegenerative diseases represent a common disease with growing mortality and morbidity worldwide, especially in the elderly population (Gitler et al., 2017; Erkkinen et al., 2018). Alzheimer’s diseases (AD), Parkinson’s diseases (PD), Huntington’s diseases (HD), and muscular atrophy lateral disease (ALS) are the main kinds of neurodegenerative diseases (Dugger and Dickson, 2017; Djajadikerta et al., 2020). Neurodegenerative diseases commonly share similar clinical characteristics, *i.e.*, progressive functional loss of neurons in the central nervous system (CNS) or peripheral nervous system (PNS), which leads to long-term motor and/or cognitive impairments (Fang et al., 2020). Currently, few or no effective treatments are available for neurodegenerative diseases, which tend to progress in an irreversible manner and are associated with large socioeconomic and personal costs (Hou et al., 2019). Therefore, research and attention on the neurodegenerative mechanism and related prevention have considerable significance. Commonly, neurodegeneration is closely associated with progressive neuronal dysfunction and death (Elobeid et al., 2016). Many molecular pathways are implicated in the process, such as proteotoxic stress and its attendant abnormalities in ubiquitin/proteasomal and autophagosomal/lysosomal systems, oxidative stress, mitochondrial dysfunction, programmed cell death, and neuroinflammation (Dugger and Dickson, 2017; Schepici et al., 2020). Therefore, herbal treatments with multiple components have been drawing attention due to their multiple targets.

*Coreopsis tinctoria* Nutt. (CT), an annual herb in the genus *Coreopsis*, is an important traditional medicine in North America, Europe, and Asia for a long historical period (Mu et al., 2020; Shen et al., 2021). CT has a variety of pharmacological activities, such as antihyperglycemic, antihyperlipidemic, antihypertensive, antioxidant (Liu et al., 2020), anti-inflammatory (Yao et al., 2019), and hepatoprotective (Tsai et al., 2017; Tian et al., 2018). The antidiabetic effect of CT is widely acknowledged due to its obvious regulation of blood glucose. Glucose is the main substance to provide energy and sustain the normal function of the brain (Mergenthaler et al., 2013). Enhancing glucose availability and uptake could protect animals against major kinds of neurodegenerative diseases (Tang 2020). Therefore, we hypothesize a potential neuroprotective effect of CT due to its good hypoglycemic effect. CT contains various kinds of flavonoids, polyacetylenes, polysaccharides, phenylpropanoids, and phenol, which commonly contributed to a synergistic protective effect (Peng et al., 2019). Commonly, individual proteins with different but closely related functions would manifest alterations at the level of biological pathways. Therefore, rapid and effective systemic exploration for bioactive compounds of CT, as well as neuroprotective mechanisms is a great challenge. This will help to find new usage of an herb and new targets for potential therapeutic opportunities.

Network pharmacology, based on the theory of systems biology, is a new subject that analyzes the biological network and screens out the nodes of particular interest, with the aim of finding out synergic relationship of multi-targets and multi-compounds (Hao and Xiao, 2014). Network pharmacology is consistent with the systemic nature of disease occurrence and thus a useful tool for herbal study based on its characteristics of identifying compound–targets to find potential molecular mechanisms (Hopkins 2007; Yang et al., 2013; Kibble et al., 2015).

**FIGURE 1:**
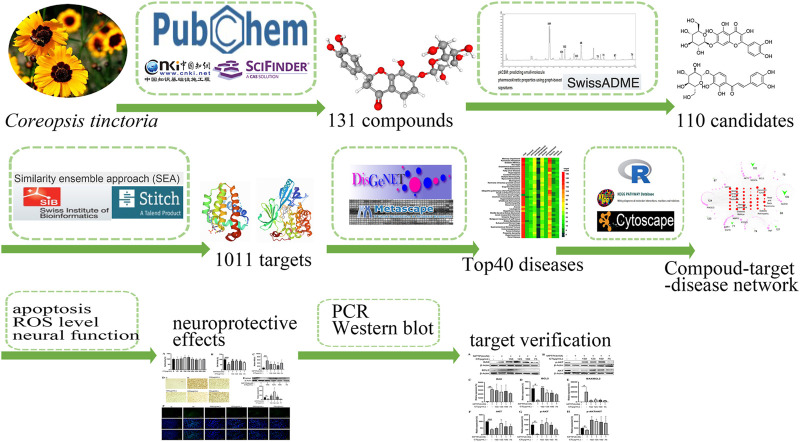
The strategy and workflow of this study.

In this study, the neuroprotective effects, the related biological pathways, and the corresponding bioactive compounds were progressively clarified. We firstly constructed database for compounds and targets. Then we predicted active compounds with suitable ADME properties and possible therapies for various disease. Thirdly, the potential mechanism for protection against memory impairment, a major predicted therapy, was further analyzed. Apoptosis was a significant characteristic during network analysis. Considering neuronal death was the typical downstream signaling for neurodegenerative diseases, for effective therapies, exploring one or more regulated upstream signaling was meaningful. Thus, finally, the neuroprotective effects and key targets for related biological pathways were confirmed in an *in vitro* model (Figure 1). This study aimed to systematically predict the molecular mechanism of active compounds of CT, while providing the potential targets in the pathway for further clinical and basis research.

## Materials and Methods

### Network Pharmacology Analysis

#### Compound Database Building

Compounds isolated from CT were mainly searched in SciFinder Scholar (https://scifinder.cas.org) with “Coreopsis tinctoria” as keywords. Their chemical information was integrated and verified by the PubChem database (https://pubchem.ncbi.nlm.nih.gov).

#### Evaluation of Drug-Likeness

Compounds with drug-likeness properties and high bioavailability are more likely to be absorbed. The predictive pharmacokinetics especially ADME (absorptions, distribution, metabolism, and excretion), bioavailability, drug-likeness, and medicinal chemistry of ligands were evaluated using the SwissADME web tool (http://www.swissadme.ch) and pkCSM approach (Pires et al., 2015). The SMILES string of each compound was input for the computational simulation. The drug-likeness was predicted using six physicochemical properties, i.e., lipophilicity (XLOGP3 between −0.7 and 5.0), size (MW between 150 and 500 g/mol), polarity (TPSA between 20 and 130 Å^2^), solubility (log S not higher than 6), flexibility (no more than nine rotatable bonds), and saturation (fraction of carbons in the sp3 hybridization not less than 0.25). The ADME properties were evaluated by passive human gastrointestinal absorption (HIA larger than 25%) and blood–brain barrier (BBB larger than −1) permeation, according to the pkCSM approach. Inhibitors for five major isoforms of cytochrome P450 (CYP1A2, CYP2C19, CYP2C9, CYP2D6, and CYP3A4) were calculated, which assessed the potential adverse effects due to low clearance. To appraise active efflux through biological membranes, interaction of compounds with the permeability glycoprotein (P-gp) was calculated.

#### Target Prediction

The putative target protein was fishing and filtered conditionally using the similarity ensemble approach (SEA) (Keiser et al., 2007), SwissTargetPrediction (Daina et al., 2019), and STITCH (Kuhn et al., 2014). At the same time, experimentally verified targets were downloaded from PubChem. Finally, target information was integrated, set to *Homo sapiens*, and aggregated for further analysis.

#### Enriched Items of Targets in the DisGeNET Database

DisGeNET is the largest publicly available dataset of genes associated to human diseases (Piñero et al., 2021). Metascape (Zhou et al., 2019) is a web-based tool for comprehensive gene annotation and analysis (http://metascape.org/). Enrichment of target genes on diseases was identified in DisGeNET using Metascape. All genes in the genome were used as the enrichment background. Disease terms with a *p*-value <0.01, a minimum count of 3, and an enrichment factor >1.5 (the enrichment factor was the ratio between the observed counts and the counts expected by chance) were collected and grouped into clusters based on their membership similarities. The *p*-values were calculated based on the accumulative hypergeometric distribution, and *q*-values were calculated using the Benjamini–Hochberg procedure to account for multiple testing. Kappa scores were used as the similarity metric when performing hierarchical clustering on the enriched terms, and sub-trees with a similarity of >0.3 were considered a cluster. The most statistically significant term within a cluster was chosen to represent the cluster.

All targets were identified in DisGeNET, and the related compounds were reversely matched. Then the compounds were classified into phenylpropanoids, phenols, polyacetylenes, terpenoids, steroids, alkaloids, phenylethanoid glycosides, anthraquinones, and flavonoids. Target information for each type was calculated and drawn in Circos and heatmap using R software.

#### Construction of the Compound–Target–Disease Network

To observe an overall relationship between compounds and their potential targets and related diseases, network analysis was performed.

The top 40 diseases in DisGeNET were screened according to enrichment significance and matched with targets in each enriched disease (logP < −70 and GeneInGOAndHitList > 100). Matched targets, related compounds, and these diseases were constructed for the compound–target–disease network using Cytoscape 3.8.2. Ten compounds with relatively high content in CT were extracted for a further compound–target–disease network analysis.

#### Gene Ontology and KEGG Enrichment Analyses

There are three sub-analyses for Gene Ontology (GO): GO cellular components (CC), GO molecular functions (MF), and GO biological processes (BP). Enrichment analyses of GO and Kyoto Encyclopedia of Genes and Genomes (KEGG) were implemented by the enrichplot package in R 3.6. Terms with a corrected *p* <0.05 were significantly enriched. The top 15 enriched items were represented with a bubble diagram via R.

### *In Vitro* Experiments Using SH-SY5Y Cells

#### Extraction of *Coreopsis tinctoria*

The CT extract was previously prepared (Yu et al., 2019). Briefly, dried flowers (100 kg) were purchased from Xinjiang, confirmed by Prof. Sibao Chen at the Institute of Medicinal Plant Development, and extracted by 80% methonal three times. The condensed extract was then fractionated by HP-20 resin, and the 70% ethanol eluent was concentrated as the extract of CT. Ten compounds, 2*R*/*S*-3′,4′,8-trihydroxyflavanone-7-*O*-glucoside, 4-*O*-β-d-glucopyranosyl-*p*-coumaric acid methyl ester, quercetagetin-7-*O*-β-d-glucoside, isookanin, quercetin-7-*O*-β-d-glucopyranoside, marein, coreopsin, okanin, eriodictyol, and butein, were previously isolated from CT. Their contents in the extract were quantified by our previous study (Yu et al., 2019) with purchased standards (Jiangsu Yongjian Pharmaceutical Technology Co. Ltd., China).

#### Cell Culture

Human neuroblastoma SH-SY5Y cells (Cell Resource Center, IBMS, CAMS/PUMC, China) were cultured in DMEM containing 10% fetal bovine serum (FBS, Gibco, United States) and antibiotics (100 μg/ml streptomycin and 100 U/ml penicillin, Gibco, United States) at 37°C under humidified conditions of 95% air and 5% CO_2._

#### Cell Viability Assay

Cell viability was determined by colorimetric assay using MTT (Sigma, United States). SH-SY5Y cells (2.5 × 10^5^/ml) were seeded into 96-well plates and allowed to adhere for 24 h. Cells were pretreated with the CT extract (25–400 μg/ml) for 4 h and then stimulated with MPTP (4 mM, Sigma, United States) for 20 h. Cells were incubated with MTT (final concentration: 1 mg/ml) for 4 h. The colored formazan was dissolved in DMSO (Beyotime, China) and measured at 570 nm using a microplate reader.

#### Terminal Deoxynucleotidyl Transferase-Mediated dUTP Nick End-Labeling Assay

Cell apoptosis was measured using Colorimetric TUNEL Apoptosis Assay Kits (Beyotime, China). Cells were washed with PBS twice, fixed by 4% paraformaldehyde, immersed by 0.1% Triton X-100, and blocked by endogenous peroxidase blocking solution. The slides were then washed, incubated with a mix solution composed of the enzyme terminal deoxynucleotide transferase (TdT) and biotinylated (Bio-16) dUTP in TdT, and stopped with a stop buffer. Then slides were incubated with a streptavidin–HRP conjugate, stained with DAB buffer, washed, and observed via microscope.

#### Measurement of Intracellular ROS Level

The formation of intracellular ROS was measured using a fluorescent probe, 2′,7′-dichlorofluorescein-diacetate (DCFH-DA), in a Reactive Oxygen Species Assay Kit (Beyotime, China). After 24 h of incubation, cells were incubated with DCFH-DA working buffer for 30 min. The fluorescence intensity was captured with a fluorescence microscope and determined using ImageJ software (National Institutes of Health, United States).

#### Quantitative Real-Time PCR

Total RNA was isolated from cells using TransZol Up reagent (TransGen Biotech, Beijing) according to the manufacturer’s instructions and measured for the concentration and quality by a NanoDrop ND-1000 spectrophotometer (Implen, United States). Then 500 ng of total RNA was reversely transcribed into cDNA using the TransScript One-Step gDNA Removal and cDNA Synthesis SuperMix Kit (TransGen Biotech, Beijing) by following the instructions. Real-time PCR amplification was carried out using specific primers (Table 1) and the PerfectStart® Green qPCR SuperMix Kit (TransGen Biotech, Beijing) on the Bio-Rad CFX96 Touch PCR system (Bio-Rad, United States). GAPDH was used as an internal reference gene, and fold change was calculated using the 2^−ΔΔCt^ method.

**TABLE 1:** Primer pairs for genes detected by real-time PCR.

GAPDH	Forward	GTC​AAC​GGA​TTT​GGT​CTG​TAT​T
	Reverse	AGT​CTT​CTG​GGT​GGC​AGT​GAT
AKT	Forward	TGA​CCA​TGA​ACG​AGT​TTG​AGT​A
	Reverse	GAG​GAT​CTT​CAT​GGC​GTA​GTA​G
Caspase3	Forward	CCA​AAG​ATC​ATA​CAT​GGA​AGC​G
	Reverse	CTG​AAT​GTT​TCC​CTG​AGG​TTT​G
BCL2	Forward	GAC​TTC​GCC​GAG​ATG​TCC​AG
	Reverse	GAA​CTC​AAA​GAA​GGC​CAC​AAT​C
BAX	Forward	CGA​ACT​GGA​CAG​TAA​CAT​GGA​G
	Reverse	CAG​TTT​GCT​GGC​AAA​GTA​GAA​A
PI3K	Forward	CGG​TGA​CTG​TGT​GGG​ACT​TAT​TGA​G
	Reverse	TGT​AGT​GTG​TGG​CTG​TTG​AAC​TGC
HIF1A	Forward	CCA​TTA​GAA​AGC​AGT​TCC​GCA​AGC
	Reverse	GTG​GTA​GTG​GTG​GCA​TTA​GCA​GTA​G

#### Western Blot Analysis

After 24 h of incubation, cells were lysed with RIPA (CWBIO, China), which contained a protease inhibitor cocktail and phosphatase inhibitor, and centrifuged at 12,000 r/min for 20 min. The protein concentration was measured with a BCA assay kit (CWBIO, China). Samples (20 μg) were separated by 8% SDS-PAGE gel with a protein marker (CWBIO, China), transferred to a PVDF membrane (Millipore, United States), blocked with 5% milk, and washed with TBST. The PVDF membranes were incubated with primary antibodies AKT (#4691), p-AKT (#4060), BCL-2 (#3498), BAX (#2772), BDNF (ab108319), and β-ACTIN (ab8226) overnight (Cell Signaling Technology, or Abcam, United States) and indicated with horseradish peroxidase-conjugated secondary antibodies (CWBIO, China). Then, protein bands were detected using an ECL kit (CWBIO, China) by a Bio-Rad imaging system.

#### Statistical Analysis

Statistical analysis was performed using one-way ANOVA in Prism software (GraphPad, La Jolla, CA), with Dunnett’s multiple-comparison test. Data are expressed as mean ± standard deviation (SD). A *p*-value less than 0.05 was considered statistically significant.

## Result

### Compound Collection and Screening

**FIGURE 2:**
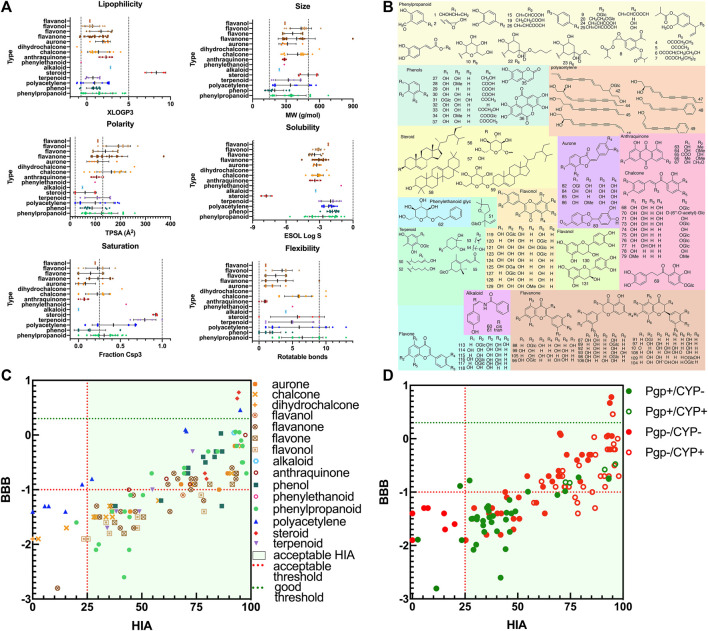
The drug-likeness properties and structures of compounds in CT. **(A)** Six physicochemical properties for evaluation of bioavailability (lipophilicity, size, polarity, solubility, flexibility, and saturation). **(B)** The 2D molecular structures of candidate compounds, **(C)** ADME properties (HIA and BBB), and **(D)** ADME properties (substrates for P-gp or CYP isoforms).

There were a total of 131 ingredients isolated from CT and identified according to literature in SciFinder Scholar. Their name, SMILES structure, and PubChem ID were calibrated using the PubChem database (Supplementary Table S1). Their predicted pharmacokinetics are shown in Supplementary Table S2. The pharmacokinetic parameters including six physicochemical properties to predict drug-likeness, HIA and BBB permeation, and interaction with P-gp/cytochrome P450 (CYP) are shown in Figures 2A,C,D. There were 110 candidate compounds for further study, with HIA > 25% and bioavailability > 0.15. They were divided into nine categories: 17 phenylpropanoids, 11 phenols, seven polyacetylenes, six terpenoids, four steroid, two alkaloids, one phenylethanoid, five anthraquinones, and 57 flavones (11 chalcones, 24 flavanones, six flavones, nine flavonols, two flavanols, and five aurones) (Figure 2B; Supplementary Table S2).

### Target Prediction and Enriched Items of Related Diseases

**FIGURE 3:**
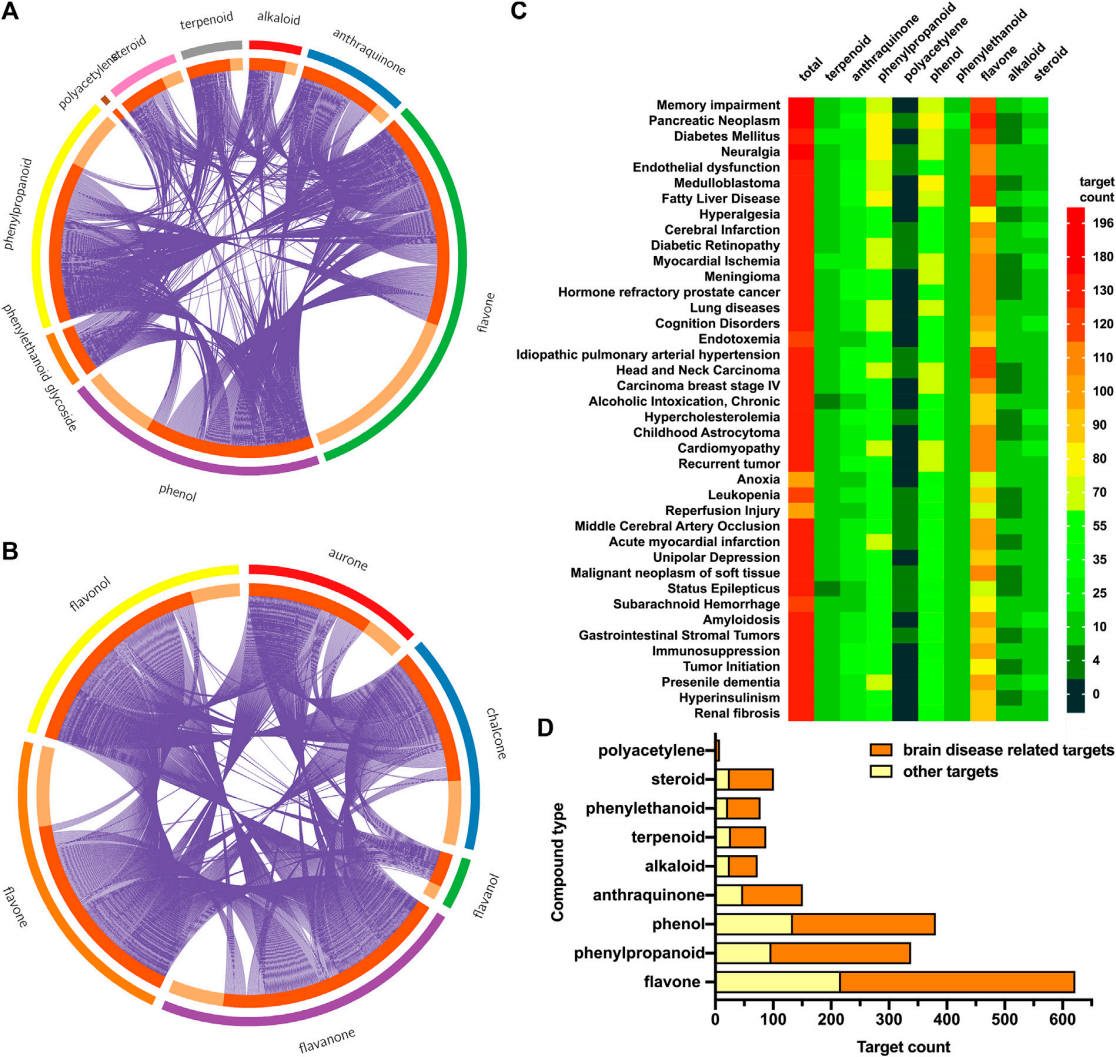
Target prediction and enrichment analysis in DisGeNET for each kind of compounds. **(A)** The distribution and overlap of targets for all kinds of compounds. Outer circle: different kinds of compounds; inner circle: predicted targets for this kind of compounds; purple line: the same target shared by different kinds of compounds. **(B)** The distribution and overlap of targets for different kinds of flavones (chalcone, aurone, flavanone, flavone, flavonol, and flavanol). **(C)** Top 40 enriched diseases in DisGeNET for different kinds of compounds. Color bar represented target gene counts. **(D)** Total targets for each kind of compounds, as well as targets enriched in items of brain-related diseases.

A total of 1,011 protein targets were predicted for 110 candidate compounds (Supplementary Table S3). Compounds in different categories shared overlap between protein targets. Most targets were regulated by flavones, phenylpropanoids, and phenols (Figure 3A). The different kinds of flavones regulated different target datasets as well (Figure 3B). Among them, flavanone, flavone, and chalcone regulated most of the protein targets. For targets of all compounds and each category, the enriched top 40 diseases in DisGeNET were also analyzed. In Figure 3C, all 110 candidate compounds had synergistic effects on memory impairment, pancreatic neoplasm, fatty liver disease, and so on. The flavones and phenylpropanoids contributed most of the synergistic effects, as they had the most enriched amounts of their targets for each disease. In addition, targets enriched in items of brain-related diseases were summarized (Figure 3D). All groups of compounds had effects on brain-related diseases. For each group, more than two-thirds of targets contributed to brain protective effects.

### Compound–Target–Disease Network Analysis for All Candidate or Main Compounds in CT

**FIGURE 4:**
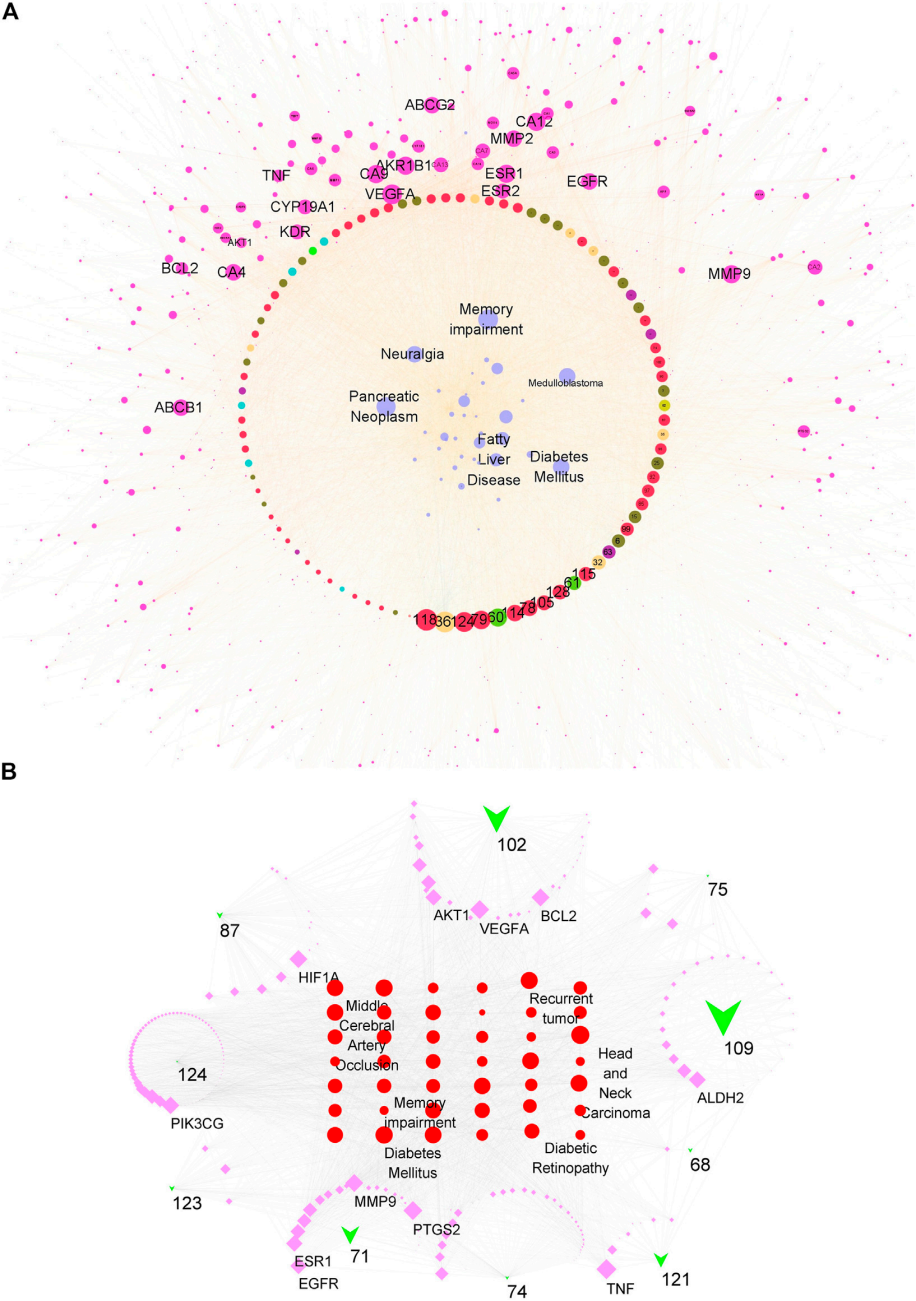
Compound–target–disease network for all candidate or main compounds of the extract of CT. **(A)** Network for all candidate compounds. The red/wathet blue/green yellow/grass green/green/blue/green/nacarat/orange/sepia node respectively represented the flavone/anthraquinone/anthraquinone/alkaloid/steroid/erpenoid/polyacetylene/phenol/phenylpropanoid compounds, the purple node represented the compound targets, wathet node represented the diseases, and edge represented the interaction between each other. **(B)** Network for 10 main compounds. The green trilateral node represented compounds, the purple square represented targets, and the red circle represented diseases.

The overall synergistic effects are firstly predicted in Figure 4A. The compound–target–disease network consisted of 1,161 nodes (110 compound nodes, 1,011 target nodes, and 40 disease nodes) and 11,585 edges in total. The purple node represented targets, the wathet blue node represented diseases, and other colors represented compounds. The edge represented the interaction between nodes. The size of each node represented its importance (degree) for this network. The larger the size, the stronger the interaction with other nodes. Most of the top important compounds belonged to flavones, showing their important role in the top 40 diseases. The node sizes of VEGFA, ESR1, MMP9, AKR1B1, CA12, CA9, ABCB1, ABCG2, BCL-2, and AKT were larger, showing that these proteins had potential to treat diseases such as memory impairment, pancreatic neoplasm, diabetes mellitus, and fatty liver disease.

The main compounds in the CT extract were determined according to our previous study (Supplementary Table S4). Nine flavonoids contributed to 69.999% of the extract content and thus were considered as the main components. Besides, quercetin, which was previously reported as a main constituent, was also included for further network analysis (Tu’erhong et al., 2016). The contribution of the dose–response relationship was worth considering for the compound–target–disease network. Therefore, we further constructed a network consisting of 10 compounds (Figure 4B). The node size of a compound was calculated via multiplying the degree of network contribution and content ratio. This network consisted of 278 nodes (10 compound nodes, 238 target nodes, and 40 disease nodes) and 2,726 edges. The green trilateral node represented compounds, the purple square represented targets, and the red circle represented diseases. Nodes with a larger size had a closer relationship with other nodes and were more important for this network. The most affected diseases were head and neck carcinoma, diabetes mellitus, fatty liver disease, memory impairment, and middle cerebral artery occlusion. Targets which were mostly regulated by the 10 compounds were TNF, PTGS2, VEGFA, BCL2, HIF1A, MMP9, PIK3CG, ALDH2, AKT1, and EGFR. Considering contributions of content and interaction with targets, the three most important compounds were 109 (isookanin-7-*O*-β-d-glucoside), 102 (isookanin), and 71 (okanin). The perturbation pattern of the top target-disease regulated by either the total or 10 compounds shared luster similarity; we concluded that the 10 compounds with high content contributed a pivotal role for the regulatory effects of the CT extract.

### GO and KEGG Enrichment Analyses

**FIGURE 5:**
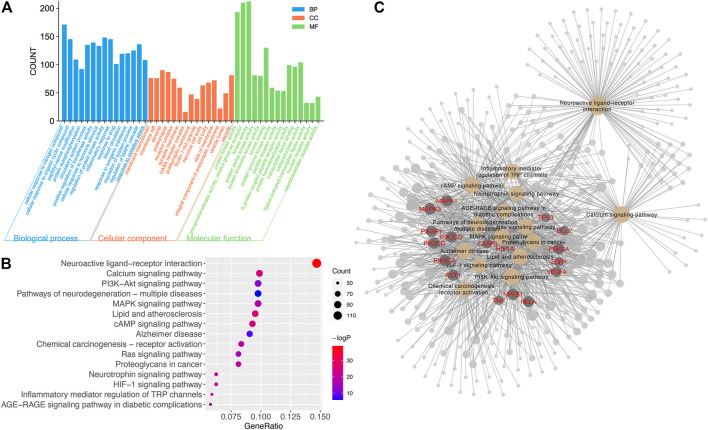
The result of GO and KEGG analyses. **(A)** The distribution of GO entries in BP, MF, and CC (top 15 according to FDR < 0.05). **(B)** Top 15 enriched KEGG items of predicted targets (FDR < 0.05). **(C)** Enriched targets in top 15 KEGG items. The yellow node represented KEGG items. The dark gray node with red gene name represented important targets. The node with a larger size contributed more to the network.

Enriched items of GO (BP, CC, and MF) and KEGG pathways for all targets were calculated (Figure 5). The top 15 of the total 4,679 GO entries, according to *p*-value and count of enriched genes, are shown in Figure 5A. There were 3,856 entries related to BP, including cellular response to nitrogen compound, peptidyl-serine modification, circulatory system process, inflammatory response, response to oxidative stress, and so on. There were 591 items related to MF, including protein kinase activity, kinase binding, G protein-coupled peptide receptor activity, oxidoreductase activity, neurotransmitter receptor activity, and so on. There were 232 CC entries, including membrane microdomain, dendrite, post synapse, neuronal cell body, and so on. Many enriched GO items were previously reported to be closely related to neuroprotection; thus, further experiments were valuable in exploring the relationship between active ingredients in CT and their biological pathways.

There were 232 significantly enriched KEGG items with *p* < 0.05. The top 15 pathways with the most significant *p*-value and largest count of enriched genes are shown in Figure 5B, including the neuroactive ligand–receptor interaction, cAMP signaling pathway, calcium signaling pathway, HIF-1 signaling pathway, PI3K-AKT signaling pathway, neurotrophin signaling pathway, and so forth. Many target proteins participated in more than one pathway, as shown in Figure 5C. Among the top targets regulated by compounds in CT, PI3K, AKT, MAPKs, caspase3, VEGFA, BCL2, and TNF were important genes involved in the top 15 pathways. And these 15 pathways contributed a lot to neuronal function and merit further experimental confirmation.

### CT Extract Inhibited MPTP-Induced Apoptosis in SH-SY5Y Cells

**FIGURE 6:**
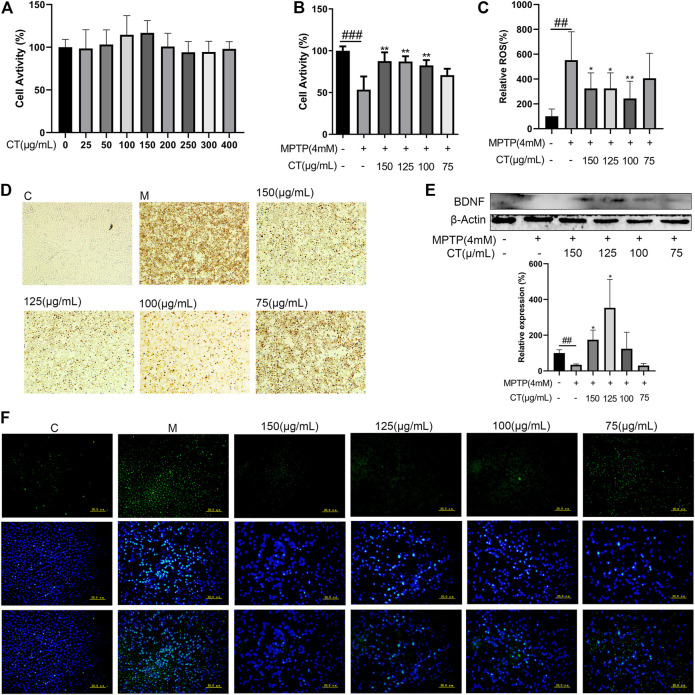
Neuroprotective effects of the CT extract. **(A,B)** Protective effects of CT on cell viability of SH-SY5Y without or with MPTP stimulation. **(C,F)** Effects of CT on intracellular ROS level. **(D)** Effects of CT on cell apoptosis by TUNEL assay. **(E)** Effects of CT on protein expression level of BDNF in MPTP-induced SH-SY5Y cells. Data were shown as mean ± SD (*n* = 3). ^#^ *p* < 0.05, ^##^ *p* < 0.01, ^###^ *p* < 0.001 when compared with the control group. ^*^ *p* < 0.05, ^**^ *p* < 0.01 when compared with the MPTP-induced model group.

Cell apoptosis and death were important stages for neuronal injury and subsequent functional loss. Here, we observed that the CT extract did not affect normal cell viability (Figure 6A). The 4 mM of MPTP significantly inhibited cell activity (53% of control), which could be significantly reversed by treatment with the CT extract in a dose-dependent manner (*p* < 0.01 for 100–150 μg/ml, Figure 6B). The CT extract could significantly restore cell activity to 87.628% of control at 150 μg/ml. The result of TUNEL assay also indicated that the CT extract remarkably reversed neuronal apoptosis, which was induced by MPTP (Figure 6D).

### CT Extract Ameliorated ROS Generation Induced by MPTP in SH-SY5Y Cells

The neurotoxicant MPTP induced neuronal apoptosis, which was accompanied by increased intracellular ROS. As Figures 6C,F have shown, the intracellular ROS level in MPTP-induced SH-SY5Y cells increased markedly to 5.52-fold of that in the control. Pretreatment of the CT extract significantly attenuated MPTP-induced ROS level (3.24-fold of that in the control at 150 μg/ml).

### CT Extract Improved the Protein Level of BDNF in MPTP-Induced SH-SY5Y Cells

BDNF was a well-studied growth factor that regulated neuronal maturation and function, synapse formation and function, motor performance, and so on (Björkholm and Monteggia, 2016; Palasz and Wysocka, 2020). Thus, the effect of the CT extract on BDNF expression was further assessed (Figure 6E). MPTP inhibited the protein expression level of BDNF (*p* < 0.01), which could be significantly reversed after CT pretreatment. Therefore, the above results suggested that the CT extract had a neuroprotective effect via ameliorating neuronal apoptosis, relieving the intracellular ROS level, and upregulating the expression level of BDNF. These were in accordance with previous network analysis and hypothesis. And as far as we know, this was the first report regarding the *in vitro* neuroprotective effect of the CT extract.

### CT Extract Regulated Gene and Protein Expression Levels of Key Targets

**FIGURE 7:**
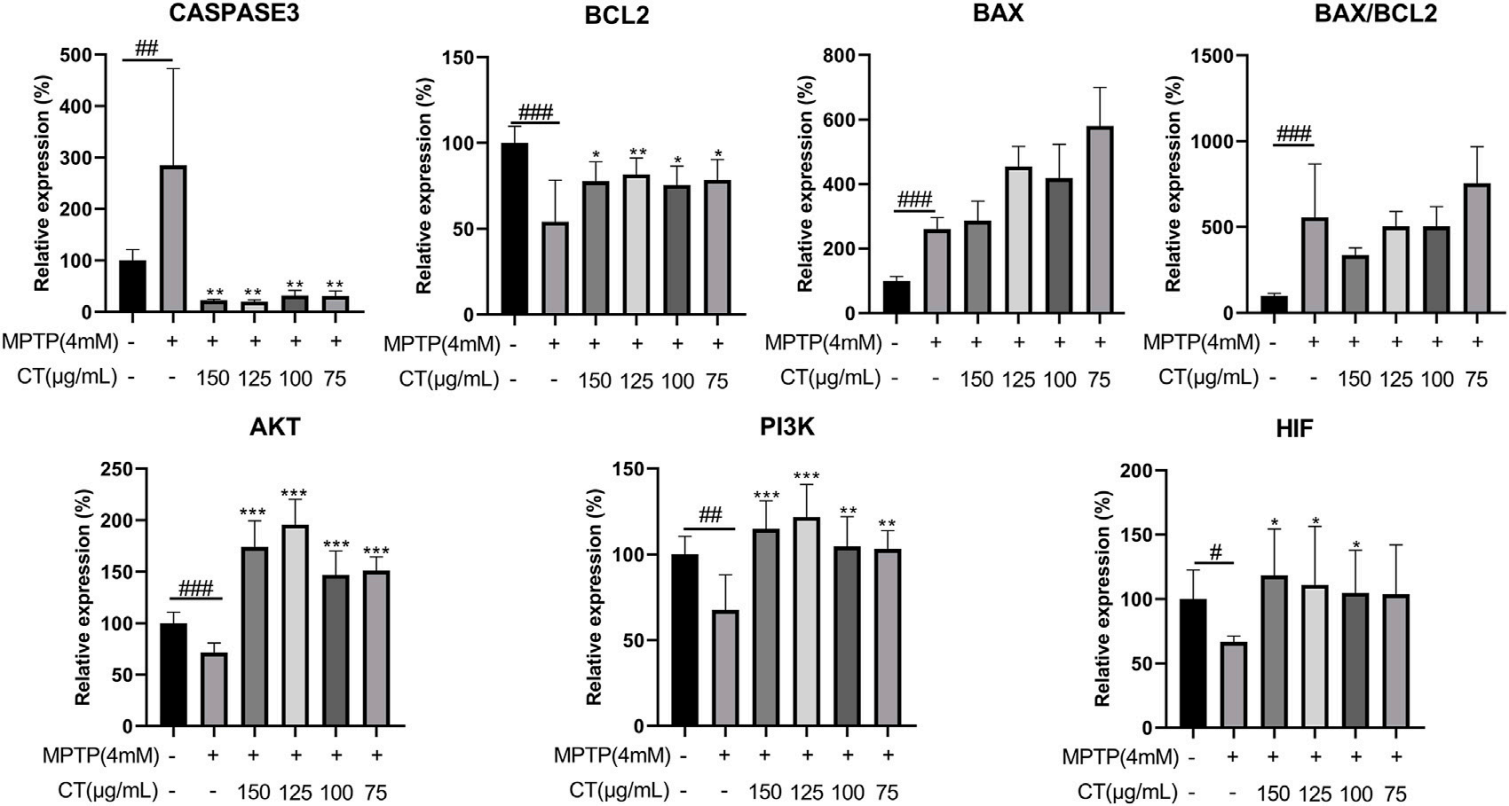
Gene expression levels of key predicted targets in cells treated with the CT extract. Data were shown as mean ± SD (*n* = 3). ^#^ *p* < 0.05, ^##^ *p* < 0.01, ^###^ *p* < 0.001 when compared with the control group. ^*^ *p* < 0.05, ^**^ *p* < 0.01, ^***^ *p* < 0.001 when compared with the MPTP-induced model group.

According to the target–disease perturbation pattern of either the total or 10 compounds, targets regarding apoptosis and energy metabolism were mainly regulated by the CT extract, which had an important role in neuroprotective effects. Thus, gene expression levels of six core predicted targets were assessed (Figure 7). Key regulatory genes during apoptosis were caspase3, BCL2, and BAX. The CT extract could significantly reduce the expression of caspase3, while obviously increasing the expression of BCL2 (*p* < 0.01 or 0.05 at 150 μg/ml). After CT treatment, the BAX/BCL2 ratio showed a decreasing trend with no significance. AKT, PI3K, and HIF-1 were the three targets regulating energy metabolism. Among them, AKT was involved in almost all top 15 KEGG pathways, such as cAMP signaling and AGE-RAGE signaling. HIF-1 was related to HIF-1 signaling, and PI3K was related to PI3K-Akt signaling. The CT extract also upregulated the expression levels of AKT, PI3K, and HIF-1 (*p* < 0.01 or 0.05 at 150 μg/ml).

**FIGURE 8:**
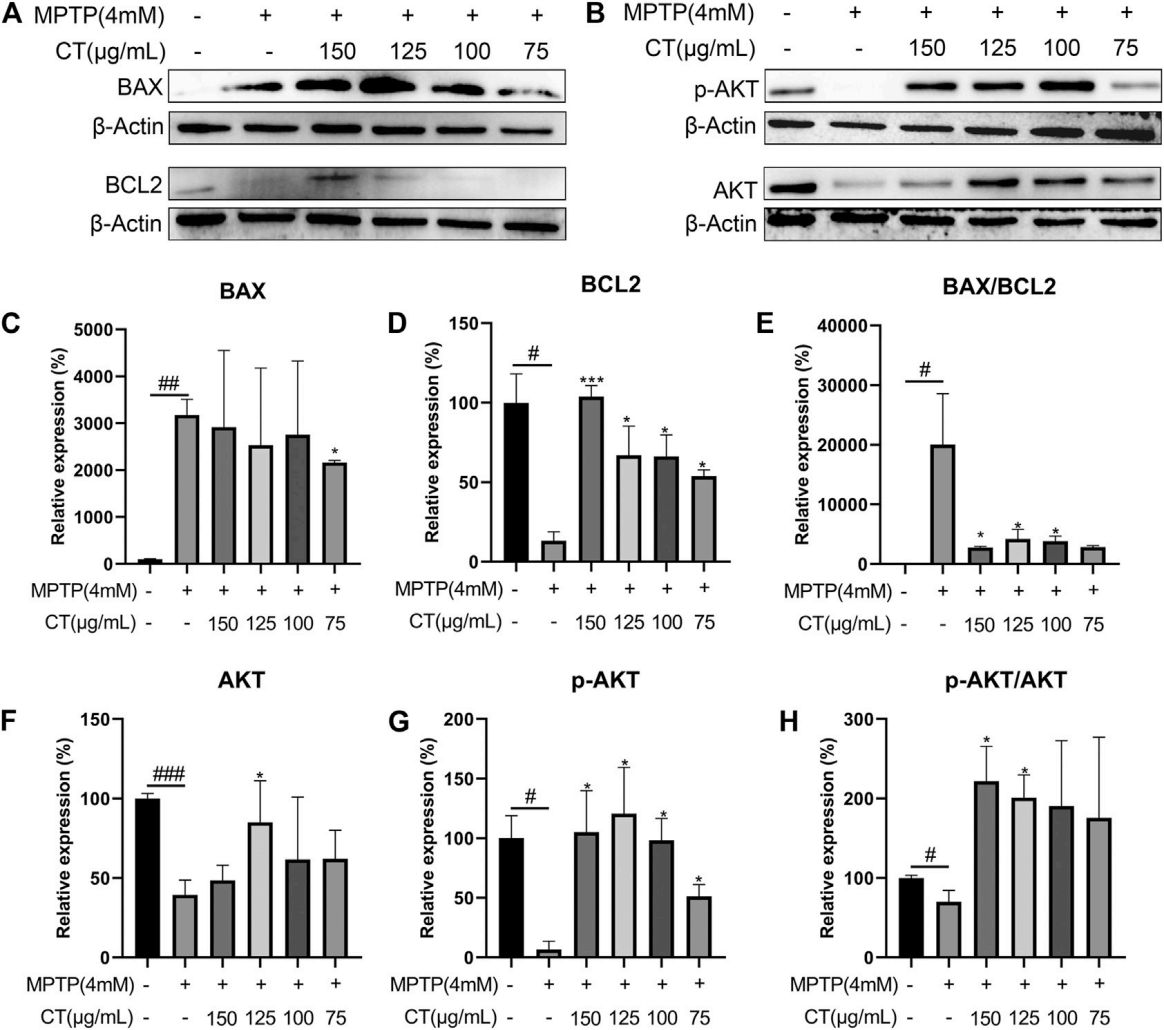
Protein expression levels of BAX, BCL2 **(A,C**–**E)**, pAKT, and AKT **(B,F–H)** in cells treated with the CT extract. Data were shown as mean ± SD (*n* = 3). ^#^ *p* < 0.05, ^##^ *p* < 0.01, ^###^ *p* < 0.001 when compared with the control group. ^*^ *p* < 0.05, ^***^ *p* < 0.001 when compared with the MPTP-induced model group.

To further identify the regulatory effects of the CT extract, we measured the protein levels of BAX and BCL2 and evaluated the phosphorylation of AKT. Compared with the control group, MPTP induced an increased expression of BAX and reduced expression of BCL2, which led to a significantly increased ratio of BAX/BCL2 (Figures 8A,C–E), whereas the CT extract treatment reversed the above apoptotic trend. As for AKT, MPTP significantly inhibited its expression and phosphorylation (Figures 8B,F–H, *p* < 0.01 or 0.05). The phosphorylation ratio of AKT (p-AKT/AKT) was significantly reduced as well. The CT extract protected against the alternation of protein expression and phosphorylation of AKT, as well as the ratio of p-AKT/AKT.

## Discussion

Herbal medicines consist of abundant and complex compounds. Network pharmacology, an approach for drug design that encompasses systems biology, network analysis, connectivity, and excessiveness, is regarded as a promising method to study the complex mechanism of TCM to improve the efficiency and potential of clinical drug development (Hopkins 2008; Pan et al., 2020). Although the development model of one-drug, one-target medicines has achieved great success, its limitations have become a reality with increased toxicity and resistance of clinical drugs. Meanwhile, some diseases such as cancer, neurological disorders, and cardiovascular diseases result from the involvement of multiple targets in multiple signaling pathways (Yuan et al., 2017). Therefore, this study has identified multiple components and multiple targets of CT and analyzed the potential relationship between components, targets, and diseases using a network pharmacological approach. CT could reduce the fasting blood glucose, serum triglyceride, and liver lipid levels and improve the development of glucose tolerance and insulin resistance in diabetic/obese mice (Li et al., 2014; Cai et al., 2016). Our results show synergistic effects of compounds in CT on memory impairment, pancreatic neoplasm, fatty liver disease, and so on. Several studies show a strong correlation between obesity/diabetes and neurodegenerative disease. Metabolic abnormalities affect neurons in the central nervous system because glucose and lipids play irreplaceable roles in energy metabolism and cell constituents (Mazon et al., 2017).

Different neurodegenerative disorders showed multiple clinical components and epidemiology in the affected anatomical regions (e.g., the brain, spinal cord, peripheral nerves, and muscle) and in the cells involved (e.g., neurons, microglia, astrocytes, and oligodendrocytes). Recent studies confirmed that neurodegenerative diseases shared common features, which were synaptic dysfunction, misfolded protein aggregation, mitochondrial deficits and ROS, transcription and translation disruption, and finally cell loss (Fan et al., 2017; Davis et al., 2018). Early cognitive and mood symptoms are mostly due to the impaired synaptic and functional networking of affected cells. Disrupted cell functions, together with DNA damage and oxidative stress, gradually lead to programmed cell death (PCD) (Vila and Przedborski, 2003). Here, during enrichment analysis of targets for potential diseases (Figure 3), compounds had a synergistic effect on memory impairment, and more than two-thirds of the targets participated in brain-related diseases for each group of compounds. Commonly, cell death was the endpoint event of neurodegeneration. Pharmacological inhibitors preventing or reducing cell loss were promising therapeutic interventions that could probably slow down the neurodegenerative progress (Bredesen et al., 2006). Thus, we hypothesized that CT potentially had neuroprotective effects, which had not been systematically studied and reported until now. Our compound–target–disease network analysis also showed that the apoptotic marker BCL2 was a top important target. Besides, many genes regarding energy metabolism and ROS generation, e.g., AKT, PIK3CG, and HIF1A, were also regulated by the main compounds in CT (Figure 4). Thus, an MPTP-induced-SH-SY5Y cell model was used here, which represented neuronal apoptosis via inhibiting complex 1 of the mitochondrial respiratory chain and increasing the ROS level (Langston 2017). Apoptosis was a type of PCD, with cytomorphology including shrinkage, chromosome condensation, and DNA fragmentation (Chi et al., 2018). Thus, we evaluated the *in vitro* neuroprotective effect of CT regarding the following four aspects: cell viability, cell apoptosis, intracellular ROS generation, and expression of neuronal function-related protein (BDNF) (Figure 6). CT showed an *in vitro* neuroprotective effect, which was consistent with the prediction of network pharmacology. Commonly, the MAO inhibitor pargyline could significantly alleviate MPTP-induced (30 mM) neuronal apoptosis (Song et al., 1997). In recent years, other herbal extract have also been studied for their neuroprotective effects. Gagam-Sipjeondaebo-Tang (100 μg/ml) could reverse cell viability inhibited by 1 mM MPTP, which was a little weaker compared with the effect of the CT extract here (Ko et al., 2018). Therefore, we suggested that the CT extract might be a candidate for the treatment and prevention of neurodegenerative disease.

Results of network pharmacology showed that, compounds (marein, okanin, butein, coreopsin, eriodictyol, isookanin, isookanin-7-*O*-β-d-glucoside, quercetagetin-7-*O*-β-d-glucoside, quercetin-7-*O*-β-d-glucopyranoside, and quercetin) played a pivotal role via acting on TNF, PTGS2, VEGFA, BCL2, HIF1A, MMP9, PIK3CG, ALDH2, AKT1, and EGFR. The mainly affected pathways were the neuroactive ligand–receptor interaction, cAMP signaling pathway, calcium signaling pathway, HIF-1 signaling pathway, and PI3K-Akt signaling pathway. Many of the above targets and signaling pathways were confirmed in previous studies of CT. The extracts of CT could inhibit the release of proinflammatory cytokines TNF-α and IL-6 in LPS-stimulated microglial cells (Tsai et al., 2017; Zhang et al., 2019). It could ameliorate the hypertrophic H9c2 cells by decreasing the HIF-1α and PPARγ pathways, and it can inhibit calcium movements through cell membranes to treat cardiovascular disease (Sun et al., 2013; Niu et al., 2021). As for the neuroprotective effects of CT, which set of targets was involved in related pathways and responsible for this effect? The execution of apoptosis was incited by intrinsic signals, such as DNA damage, p53 activation, and the upregulation of pro-apoptotic factors in the BCL2 family (Roos and Kaina, 2006). They altered the inner mitochondrial membrane permeability, which led to the release of pro-apoptotic factors (such as cytochrome c) from the mitochondria into the cytosol. Subsequently, cytochrome c interacted with procaspase9 to activate caspase3, thus promoting the execution of apoptosis in a caspase-dependent manner (Redza-Dutordoir and Averill-Bates, 2016). AKT, belonging to the cAMP-dependent AGC (PKA, PKG, and PKC) protein kinase superfamily, was highly expressed in the nervous system and played important roles in BCL2 signaling for neuroprotection. Active phosphorylated AKT promoted neuronal cell survival by increasing the phosphorylation of downstream substrates including FOXOs, GSK3β, and caspase9 (Rai et al., 2019). In addition, AKT activation induced high expression of HIF-1, which could degrade the extracellular matrix and upregulate the expression of VEGF and other angiogenic factors, thereby promoting angiogenesis (Wei et al., 2017). Thus, important targets predicted by network pharmacology had a synergistic effect on neuroprotection. The gene and protein expression levels of these targets were also reversely regulated after CT treatment, compared with cells induced by MPTP (Figures 7, 8). Therefore, CT may act on several targets such as AKT, PI3K, and HIF through the PI3K/AKT pathway and HIF-1 signaling pathway to play a role in neuronal apoptosis. According to the network pharmacological results, compounds contributed the most to neuronal apoptosis via AKT signaling. Future studies should test the relationship between BCL-2/AKT signaling and these compounds’ treatment in MPTP models.

## Conclusion

In this study, we combined a network pharmacology analysis with *in vitro* verification to explore the neuroprotective effects of CT and its mechanism. We confirmed that CT inhibited neuronal apoptosis via multiple targets on BCL2 and AKT signaling pathways. In the future, we will do further studies to investigate the overall perturbation pattern of potential pathways and mechanisms predicted in this study.

## Data Availability

The datasets for network pharmacology can be found in online repositories. The names of the repository/repositories and accession number(s) can be found in the article/Supplementary Material. The raw data for pharmacological experiments will be made available by the authors, without undue reservation.
