# Supplementary material for: The Neuroprotective Effects of Coreopsis tinctoria and Its Mechanism: Interpretation of Network Pharmacological and Experimental Data
Source: Front Pharmacol. 2022 Feb 11;12:791288. doi: 10.3389/fphar.2021.791288 (PMC8874282; doi:10.3389/fphar.2021.791288)
Supplement: Supplementary file 1 [file DataSheet1.DOCX]

Supplementary Material

**Supplementary Figure 1.** The UPLC chromatogram of CT extract (A) and calculation method (B).


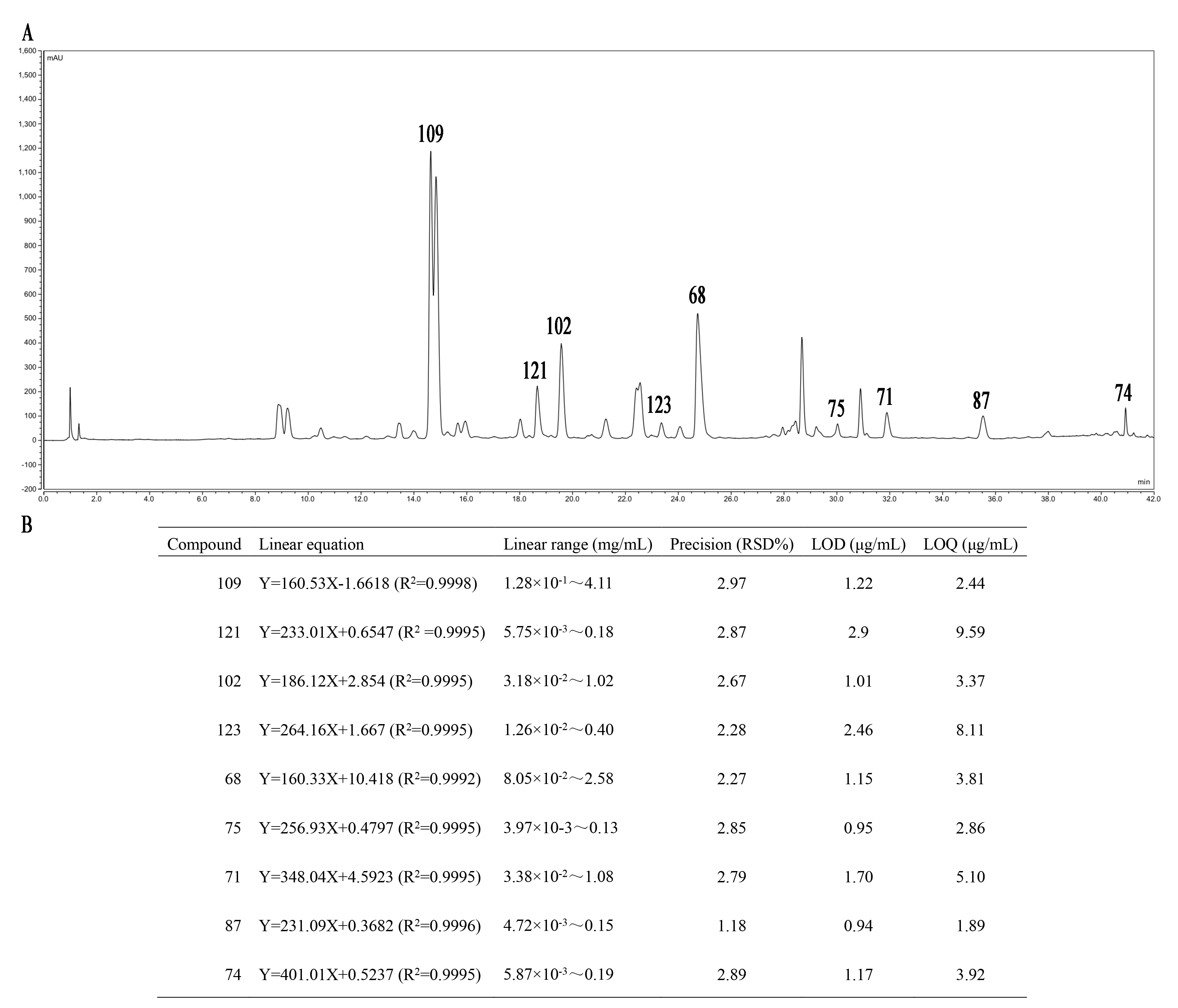


**Supplementary Table 1.** Detailed information for 131 compounds isolated from *Coreopsis tinctoria*.

**Supplementary Table 2.** Predicted pharmacokinetics for 131 compounds isolated from *Coreopsis tinctoria*.

**Supplementary Table 3.** Predicted protein targets for candidate compounds isolated from *Coreopsis tinctoria*.

**Supplementary Table 4.** Content of the main components for CT extract.

| No | Name | content in the extract (mg/g) |
| --- | --- | --- |
| 109 | isookanin-7-O-*β*-D-glucoside | 278 |
| 121 | quercetagetin-7-O-*β*-D-glucoside | 51.3 |
| 102 | isookanin | 64.8 |
| 123 | quercetin-7-O-*β*-D-glucopyranoside | 11.1 |
| 68 | marein | 230 |
| 75 | coreopsin | 7.54 |
| 71 | okanin | 48.4 |
| 87 | eriodictyol | 6.07 |
| 74 | butein | 2.78 |
